# Etiologies, diagnostic strategies, and outcomes of diffuse pulmonary infiltrates causing acute respiratory failure in cancer patients: a retrospective observational study

**DOI:** 10.1186/cc12829

**Published:** 2013-07-23

**Authors:** Hongseok Yoo, Gee Young Suh, Byeong-Ho Jeong, So Yeon Lim, Man Pyo Chung, O Jung Kwon, Kyeongman Jeon

**Affiliations:** 1 Division of Pulmonary and Critical Care Medicine, Department of Medicine, Samsung Medical Center, Sungkyunkwan University School of Medicine, Seoul, Republic of Korea; 2 Department of Critical Care Medicine, Samsung Medical Center, Sungkyunkwan University School of Medicine, Seoul, Republic of Korea

**Keywords:** cancer, respiratory insufficiency, bronchoscopy, bronchoalveolar lavage, biopsy, outcome

## Abstract

**Introduction:**

Although previous studies have reported etiologies, diagnostic strategies, and outcomes of acute respiratory failure (ARF) in cancer patients, few studies investigated ARF in cancer patients presenting with diffuse pulmonary infiltrates.

**Methods:**

This was a retrospective observational study of 214 consecutive cancer patients with diffuse pulmonary infiltrates on chest radiography admitted to the oncology medical intensive care unit for acute respiratory failure between July 2009 and June 2011.

**Results:**

After diagnostic investigations including bronchoalveolar lavage in 160 (75%) patients, transbronchial lung biopsy in 75 (35%), and surgical lung biopsy in 6 (3%), the etiologies of diffuse pulmonary infiltrates causing ARF were identified in 187 (87%) patients. The most common etiology was infection (138, 64%), followed by drug-induced pneumonitis (13, 6%) and metastasis (12, 6%). Based on the etiologic diagnoses, therapies for diffuse pulmonary infiltrates were subsequently modified in 99 (46%) patients. Diagnostic yield (46%, 62%, 85%, and 100%; *P *for trend < 0.001) and frequency of therapeutic modifications (14%, 37%, 52%, and 100%; *P *for trend < 0.001) were significantly increased with additional invasive tests. Patients with therapeutic modification had a 34% lower in-hospital mortality rate than patients without therapeutic modification (38% *versus *58%, *P *= 0.004) and a similar difference in mortality rate was observed up to 90 days (55% *versus *73%, Log-rank *P *= 0.004). After adjusting for potential confounding factors, therapeutic modification was still significantly associated with reduced in-hospital mortality (adjusted OR 0.509, 95% CI 0.281-0.920).

**Conclusions:**

Invasive diagnostic tests, including lung biopsy, increased diagnostic yield and caused therapeutic modification that was significantly associated with better outcomes for diffuse pulmonary infiltrates causing ARF in cancer patients.

## Introduction

Recent advances in the early diagnosis and aggressive management of cancer have improved long-term outcomes in cancer patients [1]. However, a number of patients with cancer may develop either malignancy-related complications or treatment-associated side effects requiring multidisciplinary care in an intensive care unit (ICU) [2]. Acute respiratory failure (ARF) is the most common cause for admission to the ICU in critically ill cancer patients and is usually associated with a poor outcome [3,4]. Although etiologies of respiratory failure in cancer patients are diverse [5], the occurrence is often associated with development of diffuse pulmonary infiltrates.

Various diagnostic strategies can be used to identify the etiologies of ARF [5]. Consequently, definitive diagnosis can be made in about 80% of cancer patients with ARF [3,6,7]. However, most of these studies on etiologies of ARF included all cancer patients with ARF, regardless of extent of pulmonary infiltrates. Therefore, data for the specific causes and outcomes of ARF presenting with diffuse pulmonary infiltrates in cancer patients are scarce in the literature. The objective of this study was to ascertain the etiologies of, diagnostic strategies for, and outcomes of diffuse pulmonary infiltrates causing ARF in cancer patients.

## Materials and methods

This was a retrospective observational study on the etiology of diffuse pulmonary infiltrates causing ARF in cancer patients admitted to the oncology medical ICU of Samsung Comprehensive Cancer Center of Samsung Medical Center (a 1,960-bed, university-affiliated, tertiary referral hospital in Seoul, South Korea) between July 2009 and June 2011; the ICU has 14 beds and provides care for approximately 350 critically ill cancer patients per year. The study was approved by the Institutional Review Board of Samsung Medical Center to review and publish information obtained from the patients' records. Informed consent was waived because of the retrospective nature of the study.

### Study population

Over the study period, a total of 583 patients with cancer were admitted to the oncology medical ICU. Of these patients, a total of 214 consecutive cancer patients with ARF and diffuse pulmonary infiltrates on chest radiography were included in the study. There was no patient with more than one ICU admission over the study period. ARF was defined as the presence of respiratory distress symptoms such as tachypnea (respiration rate >30/min) with either need for ventilator support or an arterial partial pressure of oxygen (PaO_2_)/fraction of inspired oxygen (FiO_2_) ratio (PF ratio) <300. Diffuse pulmonary infiltrates were defined as diffuse opacities involving all four quadrants of lung on chest radiography [8].

### Diagnostic evaluations

According to our diagnostic strategy for pulmonary infectious disease in cancer patients with diffuse pulmonary infiltrates, non-invasive tests including blood cultures; examination of the sputum for bacteria, mycobacteria, and fungi; urine tests for antigens of *Streptococcus pneumoniae *and *Legionella pneumophila*; and serologic tests for *Mycoplasma *were performed routinely. Blood tests for cytomegalovirus (CMV) antigen [9] and an *Aspergillus *galactomannan assay [10] were performed when there was a high suspicion of any of these pathogens. In addition, chest computed tomography (CT) scans were also performed to differentiate etiologies of diffuse pulmonary infiltrates if possible. For etiologic evaluation of acute respiratory failure from causes other than pulmonary disease, echocardiography, and blood tests for cardiac markers were performed in the ICU.

Bronchoaveolar lavage (BAL), transbronchial lung biopsy (TBLB), and surgical lung biopsy (SLB) were performed based on a joint decision of attending physicians and intensivists. BAL was performed using the standard technique as previously described [11]. BAL fluid samples were stained using Gram and Ziehl-Neelsen methods and then cultured for bacteria, mycobacteria, and fungi. Multiplex nested polymerase chain reaction (PCR) assays were used to detect influenza viruses A and B; parainfluenza viruses 1, 2, and 3; respiratory syncytial virus; and adenovirus [12]. Quantification of CMV DNA in BAL fluid was also performed using quantitative real-time PCR [13]. In addition, CMV and adenovirus were cultured using standard techniques. Calcoflour white stain and Grocott-Gomori methenamine silver stain were used to detect *Pneumocystis jiroveci *in BAL fluid [14]. TBLB and SLB were performed at the segment exhibiting the most severe abnormalities by chest CT scan. Specimens were analyzed by cultures, staining, and histologic testing.

### Definitions

Diagnoses were based on clinical, radiologic, microbiologic, and histopathologic findings [3] and were thoroughly reviewed by two of the authors (HY and KJ). Differences in observed findings were resolved by consensus. All diagnoses were classified as definite, probable, or non-diagnostic as follows. Definite diagnosis of bacterial pneumonia was defined as the pathogen being identified in BAL fluid in an amount >10^4 ^CFU/mL or the same pathogen being isolated from respiratory specimens and blood cultures. Probable diagnosis of bacterial pneumonia was defined as improvement of the patient's clinical conditions after antibiotic treatment even though the pathogen was not isolated from a respiratory specimen. Definite viral pneumonia was defined as observation of the cytopathologic effect of a virus in histopathology [15]. Probable viral pneumonia was defined as recovery of a virus in BAL fluid or nasopharyngeal specimens from a patient with clinical features consistent with viral pneumonia. However, a positive CMV PCR test or antigenemia was not sufficient for diagnosis of CMV pneumonia [3]. Pulmonary aspergillosis was diagnosed according to recently developed criteria for invasive fungal disease [16]. The diagnosis of *P. jiroveci *pneumonia was made by identification of *P. jiroveci *from a clinically relevant specimen of sputum, BAL fluid or lung tissue [17]. Cardiogenic pulmonary edema was diagnosed when echocardiography and biomarkers showed cardiac dysfunction in patients with a compatible clinical picture [18]. Diffuse alveolar hemorrhage (DAH) associated with an underlying malignancy was diagnosed using previously reported criteria [19]. However, alveolar hemorrhage was considered to be a manifestation of lung disease but not a cause of ARF, except in patients with no BAL evidence of infection and no evidence of congestive heart failure [19,20]. Drug-induced pneumonitis was defined as combination of the presence of a compatible clinical pattern, drug that is a known or suspected offender, and the exclusion of infection or pulmonary involvement from the underlying malignancy [21]. As this study included only patients with malignancies, other definitions associated with cancer status were defined by previously reported definitions [22-24]. Patients experiencing a relapse in their malignancies following intensive front-line chemotherapy or who failed to respond to initial chemotherapy were considered to be in a relapsed/refractory status [22]. The extensiveness of malignancy was classified according to the extent of the tumor and major organ involvement, as reported previously [22,23]. Disease extent was evaluated as extensive disease and/or major organ involvement. Extensive disease was defined as stage III or IV for lymphoma; metastatic or locally extensive disease for solid malignancies; and >80% blasts in bone marrow, >25,000 blasts/μL in peripheral blood, or the need for leukapheresis for hematological malignancies [22]. Major organ involvement was defined as a pathologically confirmed or radiologically suspected invasion of the brain, heart, lung, liver, or kidney [24]. Neutropenia was defined by an absolute neutrophil count (ANC) <0.5×10^3^/μL [25,26]. Therapeutic modification was defined as the addition, change of class, or withdrawal of antimicrobial agents, chemotherapy, or steroids according to identification of an etiology or exclusion of a presumptive diagnosis [27].

### Data collection

The following clinical data on ICU admission were extracted from the medical records: age, gender, type of malignancy, disease extent, recent chemotherapy administration, need for mechanical ventilation, need for renal replacement, or need for vasopressor therapy. Severity of illness was assessed by the Simplified Acute Physiology Score 3 (SAPS 3) [28] and Sequential Organ Failure Assessment (SOFA) [29]. Finally, we documented outcomes of cancer patients with ARF with diffuse pulmonary infiltrates including length of stays in ICU and hospital, ICU and hospital mortality, and 90-day mortality.

### Statistical analysis

Data are reported as numbers (percentages) for categorical variables and as medians with interquartile ranges (IQR, 25th-75th percentiles) for continuous variables. Continuous variables were compared using the Mann-Whitney *U *test and categorical variables using the Chi-square test or Fisher's exact test. To assess whether there was an association between addition of diagnostic procedures and identification rate of etiologies, the Mantel-Haenszel test was used to examine trends of diagnostic rate across additional diagnostic procedures.

The baseline characteristics, frequency of identified etiologic diagnosis and therapeutic modification based on the diagnosis were then compared between survivors and non-survivors to evaluate the effect of therapeutic modification on outcome. A multiple logistic regression model was used to adjust for potential confounding factors in the association between therapeutic modification and in-hospital mortality, as measured by the estimated odds ratio (OR) with the 95% confidence interval (CI). Variables with a *P *value < 0.20 in the univariate analysis were entered into a multiple logistic regression model where in-hospital mortality was the outcome variable of interest. The Hosmer-Lemeshow test was used to check the goodness of fit of the logistic regression. Discrimination capability was evaluated by determination of the area under the receiver operating characteristic (ROC) curve. To reduce the risk of multicollinearity, only one variable from a group of closely correlated variables was a candidate for inclusion in the final model. Kaplan-Meier estimation was used to determine the 90-day survival curves for therapeutic modification, which were then compared using the log-rank test for survival data.

All tests were two-sided, and a *P *value < 0.05 was considered significant. Data were analyzed using IBM SPSS Statistics 19.0 (IBM, Chicago, IL, USA).

## Results

The baseline clinical characteristics of the 214 cancer patients (116 with hematologic malignancies and 98 with solid tumors) admitted to the oncology medical ICU for ARF with diffuse pulmonary infiltrates on chest radiography are summarized in Table 1. Eighty-nine (42%) patients had received cancer chemotherapy within 4 weeks prior to ICU admission: median time from chemotherapy to ICU admission was 15 (11-20) days. On ICU admission, all patients had hypoxemia requiring oxygen therapy and 109 (51%) patients were mechanically ventilated (98 patients with invasive mechanical ventilation and 11 patients with non-invasive ventilation). A total of 210 (98%) patients were receiving one or more antibacterial agents at the time of ICU admission.

**Table 1:** Baseline characteristics of 214 cancer patients with acute respiratory failure and diffuse pulmonary infiltrates on chest radiography.

	Total (*n *= 214)	Hematologic (*n *= 115, 54%)	Solid (*n *= 99, 46%)	*P *value
Age (years)	60 (51-68)	56 (44-67)	62 (56-69)	0.002

Gender (male)	144 (67)	77 (67)	67 (68)	0.911

Co-morbidities	111 (52)	59 (51)	52 (53)	0.859
Cardiovascular	40	19	21	
Respiratory	25	11	14	
Hepatic	10	4	6	
Renal	4	3	1	
Diabetes	2	2	0	
Others^a^	15	12	3	

ECOG performance status (3 or more)	61 (28)	35 (30)	26 (26)	0.500

Type of malignancy				
Solid	98 (46)			
Hematologic	116 (54)			

Status of malignancy				
Relapsed/refractory	64 (30)	34 (30)	30 (30)	0.906
Extensive disease or major organ involvement	151 (71)	66 (58)	85 (86)	<0.001

Stem cell transplantation	28 (13)	28 (24)	0	
Allogenic	11	11		
Autologous	17	17		
Duration of malignancy, months	8.2 (2.9-19.4)	7.4 (2.6-18.2)	8.5 (3.1-24.0)	0.154

Clinical status on ICU admission				
Antibacterial agents	210 (98)	115 (100)	95 (96)	0.044
Recent chemotherapy prior to ICU admission within 4 weeks	89 (42)	45 (39)	44 (44)	0.487
Need for mechanical ventilation^b^	109 (51)	43 (37)	66 (67)	<0.001
Need for vasopressor support	52 (24)	28 (24)	24 (24)	0.986
Need for renal replacement therapy	19 (9)	16 (14)	3 (3)	0.005

Laboratory findings				
WBC,/μL	6,305 (2,250-12,378)	3,030 (730-7,280)	10,220 (5,330-15,670)	<0.001
ANC,/μL	3,970 (995-8,545)	1,700 (240-4,570)	7,520 (3380-12,680)	<0.001
Neutropenia (ANC <500/μL)	40 (18)	32 (28)	8 (8)	<0.001
Platelet, 10^3^/μL	112.5 (29.0-234.8)	35.0 (16.0-117.0)	187.0 (116.0-271.0)	<0.001
CRP, mg/dL	14.8 (7.4-22.3)	12.1 (5.5-19.8)	16.2 (10.4-24.0)	0.003
Procalcitonin, ng/mL	0.65 (0.19-2.85)	0.72 (0.19-3.70)	0.56 (0.17-2.29)	0.443
pH	7.444 (7.375-7.474)	7.452 (7.400-7.483)	7.433 (7.362-7.465)	0.017
NT-proBNP, pg/mL	1392 (273-4796)	2,108 (445-8,780)	940 (239 - 3424)	0.035
PF ratio	145 (105-214)	160 (109-234)	134 (101 - 181)	0.075

Severity of illness				
SAPS 3 (points)	61 (50-75)	65 (52-78)	58 (48-66)	0.002
SOFA score (points)	6 (3-8)	6 (4-9)	5 (3-8)	0.013

Length of stay in ICU (days)	6 (3-13)	5 (2-10)	7 (3-16)	0.012
Length of stay in hospital (days)	26 (15-44)	31 (17-59)	21 (12-34)	0.002
ICU mortality	64 (30)	33 (29)	31 (31)	0.765
In-hospital mortality	105 (49)	55 (48)	50 (51)	0.784
90-day mortality	138 (65)	68 (59)	70 (71)	0.087

^a^Chronic hepatitis (*n *= 4), graft-versus-host disease (*n *= 3), Grave's disease (*n *= 2), Parkinson's disease (*n *= 2), systemic lupus erythematosus (*n *= 2), aplastic anemia (*n *= 1), adrenal insufficiency (*n *= 1).

^b^Number includes both invasive and non-invasive mechanical ventilation.

ANC, absolute neutrophil count; CRP, C-reactive protein; ECOG, Eastern Cooperative Oncology Group; ICU, intensive care unit; IQR, interquartile range; NT-proBNP, N-terminal pro-brain natriuretic peptide; PF ratio, arterial partial pressure of oxygen (PaO_2_)/fraction of inspired oxygen (FiO_2_) ratio; SAPS 3, Simplified Acute Physiology Score 3; SOFA, Sequential Organ Failure Assessment; WBC, white blood cell.

All patients underwent noninvasive tests including chest radiography and microbiologic tests of blood and sputum (Table 2). Fiberoptic bronchoscopy with BAL was performed in 160 (75%) patients within a median time of 16 h (range, 2-27 h) after ICU admission. Of these patients, mechanical ventilator support was required during the procedures in 78 (49%) patients (invasive ventilation in 70 and non-invasive ventilation in 8). TBLBs were performed simultaneously with BAL in 75 (35%) patients. In six (3%) patients, SLB under general anesthesia was performed. Procedure-related complications were observed in 25 (16%) for bronchoscopic BAL ± TBLB and one (17%) for SLB. Intubation was required within 24 h after bronchoscopy in 10 of 90 (11%) patients who were not intubated before the procedures. Pneumothorax necessitating tube thoracostomy occurred in 12 patients, and major bleeding developed in one patient after TBLB. Prolonged air-leakage developed in one patient who underwent SLB. However, there were no cases of procedure-related death.

**Table 2:** Non-invasive and invasive diagnostic investigations performed.

	Patients, *n *(%)
Non-invasive tests	
Blood cultures for bacteria	214 (100)
Sputum examination for bacteria	214 (100)
Sputum examination for fungi	214 (100)
Sputum examination for mycobacteria	210 (98)
Urine *Streptococcus pneumonia *antigen	186 (87)
Urine *Legionella pneumophila *antigen	108 (50)
Serum *mycoplasma *antibody	159 (74)
Serum *Aspergillus *galactomannan assay	95 (44)
CMV antigenemia	94 (44)
Chest CT scans	209 (98)
Echocardiography	24 (11)
Invasive tests	
Fiberoptic bronchoscopy	160 (75)
BAL^a^	160
TBLB	75
Surgical lung biopsy	6 (3)

^a^Number includes the number of patients who received concomitant TBLB.

BAL, bronchoalveolar lavage; CMV, cytomegalovirus; CT, computed tomography; TBLB, transbronchial lung biopsy

After vigorous diagnostic investigations, the etiologies of diffuse pulmonary infiltrates causing ARF in cancer patients were identified in 187 (87%) patient: definite diagnosis in 82 (38%) and probable diagnosis in 105 (49%) (Table 3). The most common infectious etiology was bacterial pneumonia in 63 (29%) patients, followed by viral pneumonia in 39 (18%), fungal pneumonia in 19 (9%), *P. jiroveci *pneumonia in 14 (7%), and tuberculosis in three (1%) patients (Table 3). Common bacterial pathogen isolated from patients with definite bacterial pneumonia were *Staphylococcus aureus *(8, 29%), *Klebsiella pneumonia *(6, 21%), *Pseudomonas aeruginosa *(4, 14%), and *Acinetobacter baumannii *(4, 14%). Common viral pathogens were CMV (14, 36%), influenza A (8, 21%), adenovirus (7, 18%), and respiratory syncytial virus (6, 15%). Most fungal pneumonia was invasive pulmonary aspergillosis except for mucormycosis in one patient and disseminated candidiasis involving the lung in another patient. In cases of non-infectious etiologies, pulmonary metastasis and diffuse alveolar hemorrhage were proven in 12 (6%) and eight (4%) patients, respectively. One patient was suspected of Lipiodol embolism with lung injury after transarterial chemoembolization for hepatocellular carcinoma. One patient with AML in whom diffuse infiltrates developed 8 days after the use of all-transretinoid acid (ATRA) was diagnosed clinically with ATRA syndrome.

**Table 3:** Etiologies of diffuse pulmonary infiltrates causing acute respiratory failure in cancer patients.

Etiologies	Diagnostic probability (*n *= 214)			Hematologic (n = 115)		Solid (n = 99)	
	
	Total	Definite	Probable	Definite	Probable	Definite	Probable
Infectious etiologies	138 (64)	58 (27)	80 (37)	31 (27)	51 (44)	27 (27)	29 (29)
Bacterial	63 (29)	28 (13)	35 (16)	14 (12)	15 (13)	14 (14)	20 (20)
Viral	39 (18)	9 (4)	30 (14)	4 (3)	23 (20)	5 (5)	7 (7)
Fungal	19 (9)	4 (2)	15 (7)	2 (2)	13 (11)	2 (2)	2 (2)
*P. jiroveci*	14 (7)	14 (7)	0	10 (9)	0	4 (5)	0
Tuberculosis	3 (1)	3 (1)	0	1 (1)	0	2 (2)	0

Non-infectious etiologies	49 (23)	24 (11)	25 (12)	10 (9)	8 (7)	14 (14)	17 (17)
Metastasis	12 (6)	12 (6)	0	1 (1)	0	11 (11)	0
Diffuse alveolar hemorrhage	8 (4)	8 (4)	0	6 (5)	0	2 (2)	0
Cardiogenic pulmonary edema	10 (5)	3 (1)	7 (3)	2 (2)	5 (4)	1 (1)	2 (2)
Drugs	13 (6)	0	13 (6)	0	2 (2)	0	11 (11)
Others^a^	6 (3)^a^	1 (0)	5 (2)	1 (1)	1 (1)	0	4 (4)

Total	187 (87)	82 (38)	105 (49)	41 (36)	59 (51)	42 (42)	46 (47)

^a^Radiation pneumonitis (*n *= 3), lipiodol embolism with lung injury (*n *= 1), pulmonary alveolar proteinosis (*n *= 1), all-transretinoic-acid syndrome (*n *= 1).

**Figure 1:**
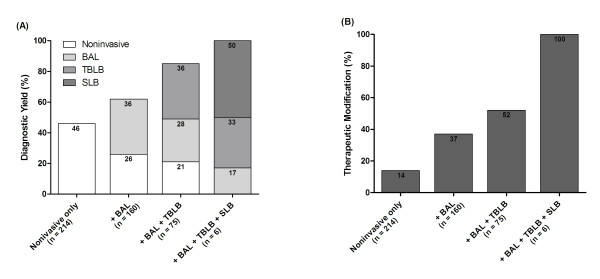
**Contributions of non-invasive and invasive tests to the diagnosis and therapeutic modifications of diffuse pulmonary infiltrates causing acute respiratory failure in cancer patients**. **(A) **Comparison of diagnostic yields across additional invasive tests in combination with non-invasive tests (*P *< 0.001). **(B) **Comparison of frequencies of therapeutic modifications across additional invasive tests in combination with noninvasive tests (*P *< 0.001).

Out of all 214 patients, the etiologies of diffuse pulmonary infiltrates causing ARF could be identified in only 99 (46%) patients through non-invasive tests. BAL exclusively provided etiologic diagnoses in 57 (36%) out of 160 patients who received BAL in addition to non-invasive tests and TBLB in combination with BAL and non-invasive tests additionally provided etiologic diagnoses in 27 (36%) patients. Etiologies were identified in all six patients with SLB providing a diagnosis exclusively in three (50%) patients. Diagnostic yield significantly increased with additional invasive tests (*P *< 0.001, test for trends) (Figure 1A). Based on the etiologic diagnoses, therapies for diffuse pulmonary infiltrates were subsequently modified in 99 (46%) patients. These modifications included initiation (*n *= 59), change (*n *= 9), or discontinuation (*n *= 15) of antimicrobial agents in 83 (84%) patients, initiation of corticosteroids in 14 (14%) patients, and initiation of chemotherapy in two (2%) patients. In addition, end-of-life decision was made based on the identified etiologies of diffuse pulmonary infiltrates in 10 patients. Frequencies of therapeutic modification based on the results from non-invasive tests and invasive tests were also evaluated across additional diagnostic tests. The frequency of therapeutic modification significantly increased with additional invasive tests (*P *< 0.001, test for trends) (Figure 1B).

**Figure 2:**
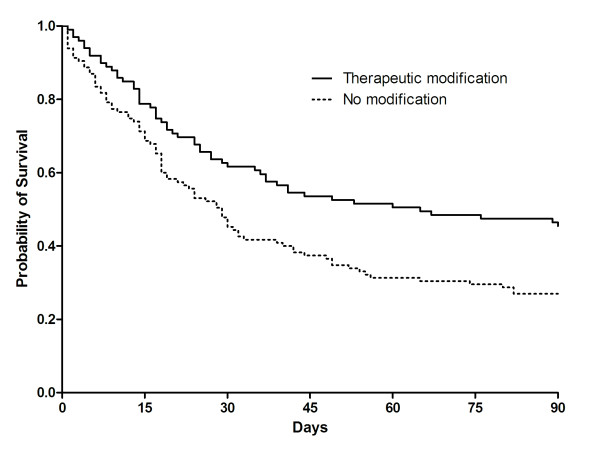
**Kaplan-Meier survival analysis comparing patients with therapeutic modification (*n *= 99) or no modification (*n *= 115) for etiologies of diffuse pulmonary infiltrates causing acute respiratory failure (*P *= 0.004, log-rank test)**.

Sixty-four (30%) patients died in the ICU after a median time of 6 days (range, 3-13 days). One hundred-five (49%) patients died during hospitalization and the median length of stay in the hospital was 26 days (range, 15-44). The 90-day mortality after ICU admission was 65%. Univariate comparisons of baseline characteristics, identified etiologic diagnosis, and therapeutic modification between survivors (*n *= 109, 51%) and non-survivors (*n *= 105, 49%) are presented in Table 4. Although there was no difference in the number of patients whose etiologic diagnoses were established (51% *vs*. 49%, *P *= 0.757), therapeutic modification based on the diagnosis of diffuse pulmonary infiltrates causing ARF was significantly associated with lower in-hospital mortality (62% *vs*. 38%, *P *= 0.004). Patients with therapeutic modification had a 34% lower in-hospital mortality rate than patients without therapeutic modification (38% *vs*. 58%, *P *= 0.004) and a similar difference in mortality rate was observed up to 90 days (*P *= 0.004, Figure 2). This finding was similar even after exclusion of 10 patients in whom the end-of-life decision was made based on the etiologic diagnosis from diagnostic strategies (see Additional file 1, Table S2/Table S3/Figure S1). After adjusting for potential confounding factors, therapeutic modification was still significantly associated with in-hospital mortality (adjusted OR 0.509, 95% CI 0.281-0.920; Hosmer-Lemeshow goodness-of-fit test, *P *= 0.325). The estimated area under the ROC curve for the therapeutic modification was 0.727 (95% CI, 0.659-0.794) (Table 5).

**Table 4:** Comparisons of baseline characteristics between survivors and non-survivors.

	Survivor (*n *= 109, 51%)	Non-survivor (*n *= 105, 49%)	Odds ratio (95% CI)	*P *value
Age (years)	59 (51-66)	62 (51-69)	1.007 (0.998-1.027)	0.477
Gender (male)	71/144 (49)	73/144 (51)	1.221 (0.689-2.165)	0.494
Co-morbidity	51/111 (46)	60/111 (54)	1.516 (0.884-2.600)	0.130
Type of malignancy				
Hematology	60/115 (52)	55/115 (48)	0.898 (0.525-1.538)	0.696
ECOG performance status (3 or more)	22/61 (36)	39/61 (64)	2.337 (1.266-4.313)	0.006
Status of malignancy				
Relapsed/refractory	24/64 (38)	40/64 (63)	2.179 (1.196-3.973)	0.010
Extensive or major organ involvement	76/151 (50)	75/151 (50)	1.086 (0.603-1.955)	0.785
Stem cell transplantation	9/28 (32)	19/28 (68)	2.455 (1.056-5.708)	0.033
Duration of malignancy, months	9.8 (3.1-22.5)	6.9 (2.7-18.4)	0.997 (0.989-1.005)	0.486
Clinical status on ICU admission				
Recent chemotherapy	88/182 (48)	94/182 (52)	2.039 (0.930-4.472)	0.071
Need for mechanical ventilation^a^	44/109 (40)	65.109 (60)	2.401 (1.386-4.157)	0.002
Need for vasopressor support	20/52 (39)	32/52 (62)	1.951 (1.030-3.695)	0.039
Need for renal replacement therapy	8/19 (42)	11/19 (58)	1.477 (0.570-3.832)	0.42
Neutropenia	12/40 (30)	28/40 (70)	2.939 (1.403-6.157)	0.003
Severity of illness				
SAPS 3 (points)	59 (50-72)	64 (50-76)	1.016 (1.001 - 1.032)	0.0420
SOFA score (points)	5 (3-7)	7 (4-9)	1.125 (1.033 - 1.226)	0.007
Identified etiology	96/187 (51)	91/187 (49)	0.880 (0.393 - 1.974)	0.757
Therapeutic modification	61/99 (62)	38/99 (38)	0.446 (0.258 - 0.773)	0.004

^a^Number includes both invasive and non-invasive mechanical ventilation.

CI, confidence interval; ECOG, Eastern Cooperative Oncology Group; ICU, intensive care unit; SAPS 3, Simplified Acute Physiology Score 3; SOFA, Sequential Organ Failure Assessment.

**Table 5:** Risk factors associated with in-hospital mortality by multiple logistic regression analysis.

	Adjusted OR (95% CI)	*P *value
Therapeutic modification	0.509 (0.281-0.920)	0.025
ECOG performance status (3 or more)	1.973 (1.027-3.791)	0.041
Stem cell transplantation	3.041 (1.242-7.447)	0.015
Need for mechanical ventilation	2.374 (1.320-4.270)	0.004
Neutropenia	2.464 (1.133-5.358)	0.023

CI, confidence interval; OR, odds ratio.

**Figure 3:**
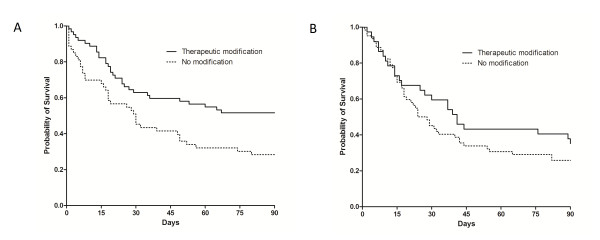
**Kaplan-Meier survival analysis comparing patients with therapeutic modification or no modification for etiologies of diffuse pulmonary infiltrates causing acute respiratory failure in patients with hematologic malignancy (A, *P *= 0.009, log-rank test) and solid tumor (B, *P *= 0.274, log-rank test)**.

Comparison of baseline characteristics according to type of malignancy is summarized in Table 1. Although extensive or major organ involvement was less frequent in patients with hematologic malignancy, severity of illness assessed by SAPS 3 and SOFA score was higher in patients with hematologic malignancy compared to patients with solid tumor. Identified etiologies of diffuse pulmonary infiltrates causing ARF are listed in Table 2. Although there was no difference in the number of patients whose etiologic diagnoses were established, the frequency of therapeutic modification based on the identified etiologies was higher in patients with hematologic malignancy compared to patients with solid tumor (Table 6). In addition, therapeutic modification based on the diagnosis of diffuse pulmonary infiltrates causing ARF was significantly associated with lower 90-day mortality in patients with hematologic malignancy (*P *= 0.009, Figure 3A). However, it was not statistically significant in patients with solid tumor (*P *= 0.274, Figure 3B).

**Table 6:** Therapeutic modification and outcomes of patients with hematologic malignancies and solid tumors.

	Hematologic (*n *= 115, 54%)	Solid (*n *= 99, 46%)	*P *value
Therapeutic modification	62 (54)	37 (37)	0.016
Initiation of antimicrobial agents	40	19	
Change of antimicrobial agents	8	1	
Withdrawal of antimicrobial agents	10	5	
Initiation of steroid	3	11	
Initiation of chemotherapy	1	1	
End-of-life decision	0	10 (10)	<0.001

Outcomes			
ICU mortality	33 (29)	31 (31)	0.677
Length of stay in ICU (days)	5 (2-10)	7 (3-16)	0.008
In-hospital mortality	55 (48)	50 (51)	0.696
Length of stay in hospital (days)	31 (17-59)	22 (12-34)	0.004
90-day mortality	68 (59)	70 (71)	0.078

ICU, intensive care units.

## Discussion

This study evaluated the etiologies of and diagnostic strategies for diffuse pulmonary infiltrates causing ARF in cancer patients admitted to the ICU. The results of our retrospective cohort study indicated that the etiologies of diffuse pulmonary infiltrates causing ARF in cancer patients could be identified in 87% of the patients after non-invasive and invasive investigations including lung biopsy. Based on the etiologic diagnoses, therapies for diffuse pulmonary infiltrates were subsequently modified in 46% of the patients. Diagnostic yield and frequency of therapeutic modification were significantly increased with the addition of invasive tests to non-invasive tests. Additionally, we found that therapeutic modification based on the etiologic diagnosis was significantly associated with lower in-hospital mortality in cancer patients with diffuse pulmonary infiltrates causing ARF.

The etiologic diagnosis of diffuse pulmonary infiltrates in cancer patients poses special challenges to the intensivist. Previous studies on the impact of diagnostic investigations including fiberoptic bronchoscopy found that the etiologic diagnoses could be identified in approximately 80% of cancer patients with ARF [3,6,7]. However, these studies could not report the etiologies of diffuse pulmonary infiltrates causing ARF in cancer patients. In addition, lung biopsy was not included in the diagnostic strategies. In this study, we included cancer patients with diffuse pulmonary infiltrates on chest radiography and performed lung biopsies in approximately 40% of the patients. As a result, bacterial pneumonia was found to be the most common cause of ARF in cancer patients with diffuse pulmonary infiltrates, consistent with that mentioned in previous reports [3,6,7]. However, viral pneumonia was also common in our cohort, which differs from previous studies. This could be associated with different nature of pulmonary infiltrates in this study and might be attributed to the fact that lung biopsy was performed. Confirmative diagnosis of viral pneumonia in this study was made by cytopathologic effect of the virus seen on histopathology. From the results of etiologic diagnosis determined by lung biopsy in bone marrow transplant recipients with pulmonary infiltrates [8], viral pneumonia was a major infectious etiology.

Both invasive and non-invasive diagnostic strategies can be used to identify the cause of pulmonary infiltrates in cancer patients with acute respiratory failure [5]. The invasive strategy relies on fiberoptic bronchoscopy with BAL which remains the cornerstone of the diagnosis of pulmonary infiltrate in nonhypoxemic cancer patients. However, previous studies on the additional diagnostic yield of invasive techniques showed that bronchoscopy with BAL establishes the diagnosis in half of the patients at best and leads to therapeutic modification in only one-third of patients [13,30]. In addition, recent multicenter cohort and randomized controlled studies found that the additional diagnostic yield of BAL in combination with noninvasive tests is relatively low [6,7]. In this study, BAL exclusively provided etiologic diagnoses in one-third of patients who underwent BAL in addition to non-invasive tests. However, TBLB simultaneously performed with BAL increased diagnostic yield, consistent with previous reports on the diagnostic yield of TBLB added to BAL in immunocompromised patients with pulmonary infiltrates [31,32]. Moreover, SLB also provided additional diagnostic yield even though a small number of patients was included. Nevertheless, invasive diagnostic procedures have potential risk of complications. In our study, procedure-related complications were observed in about 16% of patients. However, there was no procedure-related death and no difference in complication-associated outcomes between patients who underwent invasive diagnostic procedures with and without lung biopsy (see Additional file 1, Table S4). Therefore, we suggest that lung biopsy in addition to BAL could be considered as a potential invasive diagnostic strategy to identify the cause of diffuse pulmonary infiltrates in cancer patients, if feasible. However, the risk-benefit implications of these procedures need to be further evaluated.

Identifying the cause of pulmonary infiltrates with invasive test as well as non-invasive tests is associated with better outcome in hematologic patients with ARF [27,33]. This could be explained by the fact that having a specific diagnosis leads to receiving the appropriate treatment and could eliminate the toxicity of unnecessary medications. However, a recent multicenter cohort study could not find an association between specific diagnoses and outcome in cancer patient with ARF [6]. These counterintuitive results may be explained in terms of therapeutic modification based on the results from a diagnostic strategy [30]. In the present study, in-hospital (*P *= 0.757) and 90-day (*P *= 0.860) mortalities were not different between the patients with and without a specific diagnosis of diffuse pulmonary infiltrates. However, based on the results of diagnostic tests, therapies for diffuse pulmonary infiltrates were subsequently modified in half of the patients and resulted in significantly better outcomes, consistent with results from a previous study [30]. In addition, similar finding was observed in the subgroup analysis according to type of malignancy (Figure 3A/Figure 3B). Although it is possible that the better outcome was related only to the nature of the causes of diffuse pulmonary infiltrates itself or other risk factors, our data suggest that the therapeutic modifications made on a specific diagnosis would be more important, than making a specific diagnosis itself with invasive tests, in the management of cancer patients with diffuse pulmonary infiltrates causing ARF.

To fully appreciate these results, the limitations of this study must be acknowledged. First, given its retrospective nature, selection bias may have influenced the significance of our findings. Furthermore, our study was conducted at a single institution with a specialized ICU for critically ill cancer patients, which may limit the generalizability of our findings to other centers in which no experienced intensivists are available for oncological critical care. Second, the decision to perform bronchoscopic BAL and TBLB were at the physician's discretion, and not all non-invasive tests were performed routinely. More severely ill patients might not have undergone invasive tests, especially TBLB or SLB. However, we focused on the influence of therapeutic modification rather than invasive procedures on outcome. In addition, adjusted multivariable analysis served to minimize the potential for selection bias. However, the potential for bias due to an unmeasured confounder remains.

## Conclusions

The results of this study demonstrated a significant association between invasive diagnostic tests including lung biopsy and increased diagnostic yield and therapeutic modification which resulted in better outcomes in cancer patients with diffuse pulmonary infiltrates. This finding highlights the importance of the therapeutic modification based on the results of invasive diagnostic tests in the management for diffuse pulmonary infiltrates causing ARF in cancer patients. However, this observation needs to be further evaluated by a multicenter, prospective study.

## Key messages

• The etiologies of diffuse pulmonary infiltrates causing acute respiratory failure in cancer patients could be identified in the majority of the patients.

• Invasive tests including bronchoalveolar lavage and transbronchial lung biopsy in combination with non-invasive tests additionally provided etiologic diagnoses of diffuse pulmonary infiltrates.

• Therapeutic modification based on the etiologic diagnosis was significantly associated with lower in-hospital mortality in cancer patients with diffuse pulmonary infiltrates.

## List of abbreviations

ARF: acute respiratory failure; BAL: bronchoalveolar lavage; CI: confidence interval; CT: computed tomography; CMV: cytomegalovirus; ICU: intensive care unit; IQR: interquartile range; OR: odds ratio; PCR: polymerase chain reaction; SAP3: Simplified Acute Physiology Score 3; SOFA: Sequential Organ Failure Assessment; SLB: surgical lung biopsy; TBLB: transbronchial lung biopsy.

## Competing interests

The authors declare that they have no competing interests.

## Authors' contributions

HY collected and analyzed the data and drafted this manuscript. GYS contributed to the design of this study, analysis of the data, and writing of the manuscript. BHJ collected data and assisted with analyzing the data and drafting the manuscript. SYL, MPC, and OJK contributed to analysis and interpretation of data and revising the manuscript. KJ conceived and designed the study, analyzed the data, and wrote the final manuscript. All authors have read and approved the final manuscript.
